# Supplementary material for: GUI test case minimization using sequence mining
Source: PLoS One. 2026 Feb 19;21(2):e0339996. doi: 10.1371/journal.pone.0339996 (PMC12919771; doi:10.1371/journal.pone.0339996)
Supplement: Attachment — Submitted filename: review.pdf [file pone.0339996.s001.pdf]

Review:

The paper addresses the problem of minimizing regression test cases for Graphical User Interface (GUI) testing while preserving full requirements coverage. The proposed method introduces a **sequence recording technique** to capture coverage data, followed by **K-Means clustering** to group highly similar events. A search sequence then identifies a minimal representative set of test cases. Evaluation is performed using a **UI map generated in Visual Studio**, which is compared with an event map to validate coverage. Although the topic is highly relevant to both academia and industry, addressing cost and efficiency in regression testing. However there are some recommendations to further improve the manuscript.

1. It is not fully clear how the proposed sequence recording combined with K-Means differs from prior minimization approaches in the literature.
2. No reduction percentages, execution time savings, or coverage comparisons are given in the abstract, which limits the ability to judge impact.
3. Some sentences are overly long and could be broken down for better comprehension.
4. The Introduction section presents the problem context and motivation; however, it lacks sufficient citation support from relevant literature. Several claims, such as the importance of GUI testing, challenges in regression testing, and the effectiveness of test case minimization techniques, are stated without referencing prior studies or authoritative sources. Ensure that all **factual or statistical statements** are supported by credible sources.
5. The methodology should be expanded with **step-by-step procedural detail**, including algorithms, data formats, parameter settings, and specific tools/frameworks used. Clear definitions of "test case," "sequence," and "coverage" are essential for reproducibility and for assessing the validity of the approach.
6. While the paper addresses a relevant and practical problem in GUI regression testing and test case minimization, the literature review appears to lack **recent publications from the last 3–5 years**. The cited works primarily focus on earlier studies, which may not fully reflect the latest advancements in clustering-based minimization, automated GUI testing tools, or sequence-based event modeling.
