# Supplementary material for: GUI test case minimization using sequence mining
Source: PLoS One. 2026 Feb 19;21(2):e0339996. doi: 10.1371/journal.pone.0339996 (PMC12919771; doi:10.1371/journal.pone.0339996)
Supplement: Attachment — Submitted filename: Raheela revised paper.docx [file pone.0339996.s002.docx]

**Title: GUI TEST CASE MINIMIZATION USING SEQUENCE MINING**

**Supervisors:Dr Tamim Ahmed Khan & Engr.Raheela Ambrin**

| **Reviewers Comments** | **Authors Answer** | **Page Number/Section number** |
| --- | --- | --- |
| 1- Reviewer #1: The paper needs the minor revision. Please go through the comments to modify the paper accordingly. The manuscript is accepted with minor revisions. There are some grammatical and sentence structure mistakes. Besides, the methodology section should also be modified, and there have to be the in-text citations in the manuscript. Specifically, the Introduction section lacks any such support. Also the updated references are missing in the manuscript. For details, please see the comments file attached. | The entire manuscript is proofread, and all pages are revised for English language and style following reviewers’ comments. | in all pages |
| 2- Reviewer #2:   This work proposes a sequence recording technique combined with K-Means clustering to minimize GUI test cases while maintaining coverage. A medium-sized desktop application was used as a case study, where event flows, user interactions, and control usage were recorded, clustered, and analyzed for redundancy. The results show that the approach effectively reduces redundant test cases, supports regression testing, and can be extended to broader GUI testing contexts. Overall, the current work significantly improves the body of knowledge in the regression testing domain. The following are some section-wise comments to further enhance the quality of the work:   Abstract:  •It is useful to streamline the flow. Currently, the abstract is slightly long and repeats "representative set" twice. Consider condensing sentences for a sharper impact. •Example: “We propose a sequence recording technique that reports coverage and applies K-Means clustering to minimize test cases, achieving comparable requirement coverage with fewer cases.” | The manuscript is updated. The abstract is summarized, and some experimental stats are given which shows the overall percentages. | Page 1 |
| 3. Some sentences are lengthy and could be broken into two for readability (e.g., lines 8–11 about test case generation being time-consuming).  Ensure consistent terminology: sometimes you use GUI visits, GUI events, and GUI interactions. Standardize | The presentation of the manuscript is improved, and lengthy sentences are broken down.  Consistent terminologies are mentioned in the revised manuscript. | Lines 8-11 |
| 4- References [1]–[14] are summarized well but could benefit from shorter, more concise sentences. At times, the summaries are verbose (e.g., lines 83–96) | References are summarized and some recent research papers are added In the related work section | Line 103-122,272-280,281-289 |
| 5- Methodology (Section 3):  Figures: Add brief descriptive captions to make them self-explanatory (e.g., Fig. 1 “Overall Research Methodology for GUI Test Case Minimization”). | All figures and tables mentioned in the paper are elaborated and completely explain the idea. | Figure 1 -326,361,383,385,391,521,525,567,633,664,683,688,698  . |
| Tables and figures: Ensure uniform formatting. Table 2 spacing and labels look slightly inconsistent. •Highlight key findings more explicitly in text instead of leaving them implied in the table (e.g., “Sequences T3–T6 show 50% coverage and can be minimized by eliminating redundant subsets”) | Table 2 spacing is justified, and the elaborated and highlighted section shows the coverage in percentages. Findings are highlighted | Page 664 |
| Add a stronger forward-looking statement (e.g., about applying the approach to web/mobile apps, which you already mentioned briefly). •Correct capitalization in “future work” → “Future work.” | Future work is summarized and concise and clear | Line 699-718 |
| • Language / Style: •Watch for minor grammar issues, e.g., o“this application is fair enough for tracking” → “this application is suitable for tracking.” o“like some of the events” → “for example, some of the events.” •Avoid informal words like fair enough, like, etc. in academic writing. • Formatting: •Check uniform font and spacing in algorithms (Algorithm 1–4). Some indentation is inconsistent. •Ensure all acronyms are defined and consistently used. | Sentences are rephrased, and overly long sentences are broken down. Informal words are avoided, and font and spacing are justified.  Keywords are also highlighted in the paper. | Line 1-4 |
